# Nanosized Complexes of the Proteolytic Enzyme Serratiopeptidase with Cationic Block Copolymer Micelles Enhance the Proliferation and Migration of Human Cells

**DOI:** 10.3390/pharmaceutics16080988

**Published:** 2024-07-25

**Authors:** Katya Kamenova, Anna Prancheva, Lyubomira Radeva, Krassimira Yoncheva, Maya M. Zaharieva, Hristo M. Najdenski, Petar D. Petrov

**Affiliations:** 1 Institute of Polymers, Bulgarian Academy of Sciences, bl.103 Akad. G. Bonchev Str., 1113 Sofia, Bulgaria; kkamenova@polymer.bas.bg (K.K.); a_prancheva@polymer.bas.bg (A.P.); 2 Faculty of Pharmacy, Medical University of Sofia, 2 Dunav Str., 1000 Sofia, Bulgaria; l.radeva@pharmfac.mu-sofia.bg (L.R.); kyoncheva@pharmfac.mu-sofia.bg (K.Y.); 3 The Stephan Angeloff Institute of Microbiology, 1113 Sofia, Bulgaria; zaharieva26@yahoo.com (M.M.Z.); hnajdenski@gmail.com (H.M.N.)

**Keywords:** nanocarriers, polymeric micelles, serratiopeptidase, RAFT polymerization, cell proliferation, cell migration, wound healing

## Abstract

In this study, we describe the preparation of the cationic block copolymer nanocarriers of the proteolytic enzyme serratiopeptidase (SER). Firstly, an amphiphilic poly(2-(dimethylamino)ethyl methacrylate)-b-poly(ε-caprolactone)-b-poly(2-(dimethylamino)ethyl methacrylate) (PDMAEMA_9_-b-PCL_35_-b-PDMAEMA_9_) triblock copolymer was synthesized by reversible addition-fragmentation chain-transfer (RAFT) polymerization. Then, cationic micellar nanocarriers consisting of a PCL hydrophobic core and a PDMAEMA hydrophilic shell were formed by the solvent evaporation method. SER was loaded into the polymeric micelles by electrostatic interaction between the positively charged micellar shell and the negatively charged enzyme molecules. The particle size, zeta potential, and colloid stability of complexes as a function of SER concentration were investigated by dynamic and electrophoretic light scattering. It was found that SER retained its proteolytic activity after immobilization in polymeric carriers. Moreover, the complexes have a concentration-dependent enhancing effect on the proliferation and migration of human keratinocyte HaCaT and gingival fibroblast HGF cells.

## 1. Introduction

Pharmaceutical nanotechnology and innovative drug-delivery systems in particular, developed for overcoming the limitations encountered in conventional dosage forms, has made tremendous progress in recent years. The use of nanosized carriers is a promising approach for obtaining desired effects of therapies by improving the pharmacokinetics and pharmacodynamics of bioactive substances. The design of nanocarriers plays an extremely important role for the successful application of many drug candidates in medical practice and for providing more precise control over the release rate and delivery into target cells/organ [1,2,3]. Polymeric nanocarriers are one of the most promising nanosized drug-delivery systems, owing to their small size, colloidal stability, low toxicity, biodegradability, low immunogenicity, and biological inertness, as well as the presence of functional groups which can be used for the electrostatic and/or covalent binding of therapeutic agents [4,5]. Such systems enable precise dosing, the protection of the drug from degradation, reduced side effects, and the sustained release of hydrophilic and hydrophobic drugs over a given period to maintain the therapeutic level of the drug [6]. Core–shell polymeric micelles, formed from an amphiphilic block of copolymers by self-assembly, are preferentially used for developing highly efficient drug-delivery systems for the treatment of many diseases [7]. The versatility of these nanocarriers and their unique structures enable the loading of a wide variety of therapeutic agents, including hydrophobic and hydrophilic low-molecular drugs, proteins, and enzymes, and small interfering RNA (siRNA), DNA, etc. [5,8,9].

Enzymes are proteins that possess catalytic activity in living organisms and regulate the rate of biochemical reactions. Enzyme-based therapeutics are an integral part of modern medicine mainly due to their high selectivity, efficiency, and safety profile [10,11,12,13,14,15]. Although enzyme therapy has many advantages compared to conventional therapeutic approaches, it remains challenging due to the short in vivo half-life, low bioavailability, poor penetration, and, in some cases, patient immune system reactions against the enzyme [10,15,16]. In recent decades, the incorporation of proteins and peptides into nanocarriers (liposomes, polymeric nanoparticles micelles, etc.) for improving their stability and bioavailability and reducing their immunogenicity and fast clearance has gained significant interest [2,3,4,5]. The main strategies for enzyme immobilization into nanocarriers involve chemical conjugation or physical bonding based on weaker reversible interactions [17,18]. In particular, the formation of polyelectrolyte complexes between oppositely charged enzymes and polymer chains is a robust and facile strategy for the preparation of enzyme-immobilized polymeric nanosystems. Using weak polyelectrolytes such as polyamines and polycarboxylates is preferred because such polymers do not induce significant changes in protein structure or activity, respectively [19,20]. PDMAEMA is a water-soluble polycation which is sensitive to changes in the temperature and pH of an environment. The charge density of PDMAEMA can be adjusted by the protonation/deprotonation of the tertiary amine moieties upon changing pH (the pKa of the amine groups is about 7.0–7.5) [21,22,23]. The reversible properties of this polymer make it one of the most popular and well-investigated polymers for the formation of polyelectrolyte complexes via electrostatic interaction with oppositely charged macromolecules, such as polyanion polymers, proteins, enzymes [24,25], and DNA [26]. Recently, we developed a model system for the transportation and delivery of genes based on nanosized PDMAEMA-b-PCL-b-PDMAEMA block copolymer micelles, which were used as templates for forming complexes with DNA via electrostatic interaction [26]. In another study, mixed polymeric micelles, comprising a biodegradable hydrophobic PCL core, a middle PDMAEMA/PEO layer, and a hydrated PEO shell, were fabricated for the delivery of insulin. The protein was immobilized into a micellar structure via complexation with the positively charged PDMAEMA chains [27]. Sentoukas and Pispas have studied the complexation ability of poly (2-[dimethylamino]ethyl methacrylate)-b-poly(hydroxypropyl methacrylate) (PDMAEMA-b-PHPMA) polymeric micelles and their quaternized counterparts QPDMAEMA-b-PHPMA with bovine serum albumin (BSA) [28] and short DNA [29] in aqueous solutions. Their investigation was mainly focused on the structure, properties, and stability of the obtained DNA/copolymer polyplexes at different ratios of the macromolecular components and different ionic strengths of the media [28,29].

Serratiopeptidase is a proteolytic enzyme which belongs to the Serralysin group of enzymes. SER is produced by non-pathogenic enterobacteria that exist in the gut of the silkworm and facilitate the destruction of the cocoon to release the silkworm. SER (EC number 3.4.24.40) is a metalloprotease enzyme consisting of 470 amino acids, with a molar mass between 45 and 60 kDa [30]. The enzyme exerts maximum activity at pH 9 and 40 °C, but it is stable at lower T in the pH range from 5 to 10. The isoelectric point of SER is between pH 5.0 and 5.5. SER can be deactivated by heating at 55 °C for 15 min [31,32,33,34]. Due to its anti-inflammatory, fibrinolytic, antiedema, and analgesic effects, SER is used in various fields of medicine, such as orthopedics, surgery, dentistry, otorhinolaryngology, etc. [35,36]. The enzyme has been reported to be used in acute or chronic inflammatory diseases of the lungs, nose, or throat, such as bronchitis, laryngitis, and sinusitis [12,37,38]. Common limits related to enzyme delivery are instability, incompatibility, low permeability through the intestinal membrane, and low bioavailability [10,15]. The development of nanocarriers for the immobilization of SER is an interesting and challenging task. A few studies describing the incorporation of SER into magnetic nanoparticles [39], chitosan nanoparticles [34], albumin nanoparticles [40] microspheres [41,42], and liposomes [43] have been reported. As far as we know, the loading of the SER into polymeric micelles has not been reported in the scientific literature yet. This fact motivated our team to develop and characterize in detail the properties of novel nanosystem based on complexes of SER and polycationic micelles, and expand the current knowledge on SER delivery using nanocarriers.

The present work describes the first results on polyelectrolyte complexes preparation by using PDMAEMA-b-PCL-b-PDMAEMA micelles and the proteolytic enzyme serratiopeptidase. The triblock copolymer was synthesized via RAFT polymerization, which allowed good control over the composition and molecular characteristics. The obtained amphiphilic copolymer self-assembled in aqueous media into core–shell micelles composed of a hydrophobic PCL core and a positively charged PDMAEMA outer shell. SER was immobilized into the shell of nanocarriers via electrostatic interactions between positively charged PDMAEMA chains and negatively charged enzyme molecules. The formation of complexes at different polymer/enzyme ratios was investigated by dynamic and electrophoretic light scattering (DLS and ELS) and UV–visible (UV–Vis) and FTIR spectroscopy. The wound-healing potential of the complexes was evaluated via the examination of the migration and proliferation of two types of cells (human keratinocytes and human gingival fibroblasts) by the scratch test.

## 2. Materials and Methods

### 2.1. Materials

Serrapeptase, Native (*Serratia* sp.), was purchased from Creative Enzyme (New York, NY, USA. The enzyme was purified by ultrafiltration with an Amicon device with Ultracel^®^ 10 kDa ultrafiltration discs (EMD Millipore Corporation, Billerica, MA, USA) and then freeze-dried. Poly(ε-caprolactone) diol (CAPA^®^ 2402, MW 4000 g mol^−1^; kindly donated by Solvay Chemicals Inc., Houston, TX, USA) was purified by precipitation in cold methanol (−30 °C), and then filtered and dried overnight under vacuum. 2-(Dimethylamino)ethyl methacrylate monomer (98%, Sigma-Aldrich, FOT, Sofia, Bulgaria) was passed through a column of activated neutral aluminum oxide (Sigma-Aldrich, FOT, Sofia, Bulgaria to remove the inhibitor. 2,2′-Azobis(isobutyronitrile) (AIBN) initiator was used after recrystallization from methanol. The solvents dichloromethane (99.8%, DCM, Sigma-Aldrich, FOT, Sofia, Bulgaria) and tetrahydrofuran (THF, HPLC grade, Fisher Chemical, Labimex, Sofia, Bulgaria) were purified before use by stirring overnight in the presence of calcium hydride (95%, Sigma-Aldrich, FOT, Sofia, Bulgaria) and subsequent distillation. 4-cyano-4-(phenylcarbonothioylthio)pentanoic (CTA), N,N′-dicyclohexylcarbodiimide (DCC, 99%), 4-dimethylaminopyridine (DMAP, 99%), 1,6-diphenyl-1,3,5-hexatriene (98%, DPH), toluene (99%), anisole (99%), dioxane (≥99.0%), and n-hexane (99%) were purchased from Sigma-Aldrich (FOT, Sofia, Bulgaria) and used as received.

### 2.2. Methods

#### 2.2.1. Synthesis of PCL-Based Macro-RAFT Agent

The synthesis of a bifunctional macro-RAFT agent was carried out by the esterification of PCL-diol with 4-cyano-4-(phenylcarbonothioylthio)pentanoic acid in methylene dichloride in the presence of DMAP and DCC. Briefly, in a 100 mL one-neck, round-bottom flask equipped with a magnetic stirring bar, PCL-diol (1 g, 0.25 mmol, 1 eq.) and CTA (0.17 g, 0.75 mmol, 3 eq.) were dissolved in 20 mL freshly distilled DCM under an inert atmosphere. After homogenization, the solution was cooled down to 0 °C by using an ice bath. DCC (0.15 g, 0.75 mmol, 3 eq.) and DMAP (0.045 g, 0.375 mmol, 1.5 eq.) were consequently added. The mixed system was stirred in an ice bath for 20 min; then, the reaction temperature was slowly raised to 22 °C. The reaction was carried out under stirring for 48 h at room temperature. Thereafter, the reaction mixture was filtered off to remove the byproduct dicyclohexylcarbodiurea (DCU). The filtrate was concentrated by rotary evaporator and precipitated into a 10-fold excess of cold methanol (−30 °C) three times to remove the unreacted CTA. The final product was obtained by filtering and drying in a vacuum oven at 40 °C for 48 h. Yield 78%.

^1^H-NMR (CDCl_3_, δ ppm): e 7.9-7.4 (m, 10 H from phenyl groups), a 4.06–4.0 (m, 70 H, -CH_2_-O-), d 2.31 (m, 70 H, -C(O)-CH_2_-), f 1.95 (m, 6H, -C(N)-C-CH_3_), b 1.65 (m, 140 H, -C(O)-CH_2_-CH_2_-CH_2_-), c 1.39–1.35 (m, 70 H, -CH_2_-CH_2_-CH_2_-).

#### 2.2.2. Synthesis of Amphiphilic PDMAEMA_9_-b-PCL_35_-b-PDMAEMA_9_ Triblock Copolymer by RAFT Polymerization

CTA–PCL–CTA (0.350 g, 0.076 mmol) and AIBN (2.4 mg, 0.014 mmol) were dissolved in anisole (6 mL) under argon in a 50 mL round-bottom flask stoppered with a septum. After that, DMAEMA (0.31 mL, 2.1 mmol) was added, and the reaction mixture was stirred at ambient temperature under argon flow for 15 min. After degassing, the flask was immersed in an oil bath at 75 °C. After 24 h, the polymerization was terminated and THF was added to dissolve the formed copolymer. The product was purified by precipitated twice in excess of n-hexane and then dried under vacuum for 48 h. The yield was 72%.

^1^H-NMR (CDCl3, δ ppm): d (ppm) = a + i 4.06–4.00 (m, 70 H, -CH_2_-O- from PCL+ m, 36 H, –O–CH_2_–CH_2_–N– from PDMAEMA), j 2.53 (s, 36 H, -CH_2_–CH_2_–N(CH_3_)), d,k 2.45–2.31 (m, 70 H, -C(O)-CH_2_—from PCL + m), 108 H, (–N(CH_3_) from PDMAEMA), h 1.86–1.75 (36 H, –CH_2_–C–CH_3_), b 1.65 (m, 140 H, -C(O)-CH_2_-CH_2_-CH_2_-), c 1.39–1.35 (m, 70 H, -CH_2_-CH_2_-CH_2_-), g 1.00–0.64 (m, 54 H, –CH_2_–C(CO)–CH_3_).

#### 2.2.3. Preparation of PDMAEMA_9_-b-PCL_35_-b-PDMAEMA_9_ Block Copolymer Micelles and Determination of Critical Micelle Concentration

The block copolymer micelles were prepared by the self-assembly of PDMAEMA_9_-b-PCL_35_-b-PDMAEMA_9_ in aqueous media. Firstly, the copolymer (10 mg) was dissolved in THF (5 mL), which is a good solvent for PCL and PDMAEMA; then, the solution was added dropwise to deionized water (10 mL) at room temperature and stirred for 30 min. Next, THF was evaporated with a rotary vacuum evaporator, thereby obtaining an aqueous micellar solution with a concentration of 1 gL^−1^.

The critical micelle concentration (CMC) of PDMAEMA_9_-b-PCL_35_-b-PDMAEMA_9_ was calculated from the UV–Vis absorption spectra of samples containing the hydrophobic dye DPH. For this purpose, to the prepared micellar solutions (2 mL; 8 different concentrations between 0.005 and 1 gL^−1^), a methanolic solution of DPH (20 μL, 0.4 mM) was added under stirring. The samples were incubated in the dark for 16 h at room temperature before recording the UV–Vis absorption spectra of DPH in the λ = 250–600 nm range at 25 °C. The CMC values were determined as the inflection points of the absorbance intensity (at 356 nm) vs. polymer concentration curve.

#### 2.2.4. Preparation of Complexes of PDMAEMA_9_-b-PCL_35_-b-PDMAEMA_9_ Micelles and SER

The complexes of polymeric micelles and serratiopeptidase were prepared at different mass ratios polymer/enzyme (from 1:0.01 to 1:10) as follows: the enzyme, dissolved in water (5 gL^−1^), was added dropwise to 1 mL of micellar dispersion (1 gL^−1^), and then water was added to a total volume of 2 mL at room temperature under stirring. The final concentration of SER was from 0.05 to 5 gL^−1^ and the final micelle concentration was 0.5 gL^−1^.

#### 2.2.5. Characterization of Polymers

^1^H-NMR spectra were recorded on a Bruker Avance-DRX 400 MHz spectrometer (Bruker Corporation, Billerica, MA, USA). The samples were dissolved at room temperature in CDCl_3_. Gel permeation chromatography (GPC) measurements were performed with a Shimadzu Nexera HPLC chromatograph (Shimadzu Corporation, Kyoto, Japan) equipped with a degasser, a pump, an autosampler, a RI detector and three PSS SDV columns (5 μm Linear M; 5 μm, 100 Å; and 5 μm, 50 Å), using tetrahydrofuran as the eluent at a flow rate of 1.0 mL/min and a temperature of 40 °C. The sample concentration was 1 mg/mL.

#### 2.2.6. Particle Size and Zeta Potential

The hydrodynamic diameter and zeta potential of the polymeric micelles and complexes with serratiopeptidase were determined by Zetasizer NanoBrook 90Plus PALS instrument (Holtsville, NY, USA), equipped with a 35 mW red diode laser (λ = 640 nm). DLS measurements were conducted at a scattering angle of 90°at 25 °C. The ζ-potential measurements were conducted at a scattering angle of 15° and the ζ-potential was calculated from the obtained electrophoretic mobility at 25 °C.

#### 2.2.7. Fourier Transforms Infrared Spectroscopy (FTIR)

The FTIR spectra of freeze-dried SER, pure polymeric micelles, and complexes were recorded with a FTIR spectrometer (IRAffinity-1, Shimadzu, Kyoto, Japan), equipped with ATR, in the range from 600 to 4000 cm^−1^.

#### 2.2.8. UV–Vis Spectrophotometry

A UV–Vis spectrophotometer (Thermo Scientific, Waltham, MA, USA) was used to record all UV–Vis spectra of aqueous solutions of SER and complexes. All samples were put in quartz cells with a path length of 1 cm.

#### 2.2.9. Enzyme Activity

The proteolytic activity of SER was determined with Sigma’s Non-Specific Protease Activity Assay [44]. This method accounts for the hydrolysis of casein by SER to tyrosine. The concentration of tyrosine was determined via UV–Vis spectroscopy (Thermo Scientific, Waltham, MA, USA) at 660 nm. The units of protease activity (one unit is defined as the micromoles of tyrosine released for 1 min under standard conditions) were determined as follows:

(1) $$\frac{\begin{array}{c} \mathrm{Units} \end{array}}{mL}=\frac{µmol\;tyrosine\;equivalents\;released\times total\;volume\;of\;assay\;(mL)}{volume\;of\;sample\left(mL\right)\times time\;of\;assay\left(minutes\right)\times volume\;used\;in\;colorimetric\;determination\;(mL)}$$

#### 2.2.10. In Vitro Drug Release Study

The in vitro drug release test was carried out in a phosphate buffer (pH = 7.4) at 32 °C. The enzyme-loaded micelles (1 mg/mL concentration of SER) were placed in the buffer and, at predetermined time intervals, the dispersion was centrifuged at 15,000 rpm for 10 min (DLAB D20212 Plus, DLAB Scientific Co., Beijing, China); the supernatant was taken for measurement and the same amount of fresh buffer was returned. The concentration of the released enzyme was determined spectrophotometrically at 275 nm (Thermo Fisher Scientific, Waltham, MA, USA). The calculations were made according to a standard curve of the enzyme in the range of 0.1–2.5 mg/mL (r > 0.9998).

#### 2.2.11. Cell Cultures

Two non-tumorigenic cell lines were used for the evaluation of the ability of nanopolymeric complexes with serratiopeptidase to potentiate cell proliferation and migration by using the scratch assay–human keratinocytes (HaCaT) and human gingival fibroblasts (HGF), both purchased from CLS GmbH (Eppelheim, Germany). HaCaT cells were maintained in culture medium DMEM (DMEM-HPA, Capricorn^®^, Ebsdorfergrund, Germany) supplemented with 10% fetal bovine serum and 4 mM L-Glutamine. HGF cells were cultured in DMEM-Ham’s 12 (Capricorn^®^, Germany), supplemented with 10% fetal bovine serum and 2 mM L-Glutamine. The cell lines were cultured under standard conditions (5% CO_2_, 37 °C, maximal humidity) in an CO_2_ incubator (Panasonic, Tokyo, Japan) and subcultured twice a week (dilution 1:8) using the cell detachment reagent Accutase^®^ (ACC-1B, Capricorn^®^, Germany) and phosphate-buffered saline (PBS, pH 7.4) (TS1101, HiMedia, Thane, India) as a washing solution.

#### 2.2.12. Cell Viability Test

The cell viability assay for the determination of the maximal non-toxic concentrations (MNC) was performed on HaCaT cells according to ISO 10993-5-2009 [45], Annex C. The protocol used is based on the MTT reduction assay [46]. Briefly, cells were seeded in 96-well plates at a density of 0.7 × 10^5^ cells/mL. After 24 h incubation in an CO_2_ incubator, the cells were treated under sterile conditions (Laminar Air Flow Telstar Bio II Advance, Barcelona, Spain) with serratiopeptidase in concentrations ranging from 0.313 to 10 µg/mL. The incubation periods were 24 and 48 h. Every sample was repeated four times. Cell viability was determined at the end of the incubation period by adding MTT dye in a final concentration of 0.05 mg/mL. The samples were kept at 37 °C for 2 h and the resulting product (violet formazan crystals) was dissolved with 2-propanol (Sigma-Aldrich, Merck, Darmstadt, Germany) after aspirating the culture medium. The absorbance was measured at λ = 550 nm (reference filter 690 nm) against a blank solution (the organic solvent) on Absorbance Microplate Reader EL-800 (Bio-Tek Instruments Inc., Winooski, VT, USA).

#### 2.2.13. Wound Assay

The wound-healing effect of SER nanocomplexes was determined via the scratch assay on HaCaT and HGF cells [47,48] with some modifications. For the assay, cells were seeded in 24-well plates at a concentration of 1.5 × 10^5^ cells/mL and incubated until the formed an adherent monolayer (24 for HaCaT to 48 h for HGF). The scratches were made using a sterile pipette tip (200 µL) and then the cells were washed twice with culture medium. Given the fact that, in conditions close to the microenvironment of a wound, the enzyme will be in contact with exudate containing proteins and growth factors, the cells were cultured with medium with FBS but in lower concentration (5%). The cells were treated with 1.25, 2.5, and 5 g/mL of complexes for 24 and 48 h (HaCaT cells) or for 48 h (HGF cells). At the end of the incubation period, the effect was observed under inverted microscope (Boeco BIB-100, Hamburg, Germany) and documented with a digital camera under 40 and 100× magnification. ImageJ software (version 1.54 g) [49] was used for the determination of the wound-healing ability for HGF via calculating the scratch closure rate as a percentage, according to the following expression:

Scratch closure rate = 100 − As × 100/A_0_, (2)

where A_0_ corresponds to the scratch area at 0 h and As corresponds to the scratch area at 24 h/48 h.

### 2.3. Statistics

The graphs representing the viability of HaCaT cells were plotted in the GraphPad Prism software version 6.0.0. Statistical analysis was performed with one-way ANOVA followed by Dunnett’s multiple comparisons test using GraphPad Prism version 6.0.0 (GraphPad Software, Boston, MA, USA).

## 3. Results and Discussion

### 3.1. Synthesis and Characterization of the Triblock Copolymer

An amphiphilic triblock copolymer, consisting of a middle poly(ε-caprolactone) hydrophobic block and two outer hydrophilic blocks of poly(2-(dimethylamino)ethyl methacrylate) (PDMAEMA), was synthesized and used for obtaining the cationic micellar nanocarriers. Poly(ε-caprolactone) is a biocompatible and biodegradable polymer often used in medicine, while PDMAEMA is a temperature- and pH-sensitive polymer which is often applied in preparing complexes with negatively charged biomolecules.

**Figure 1:**
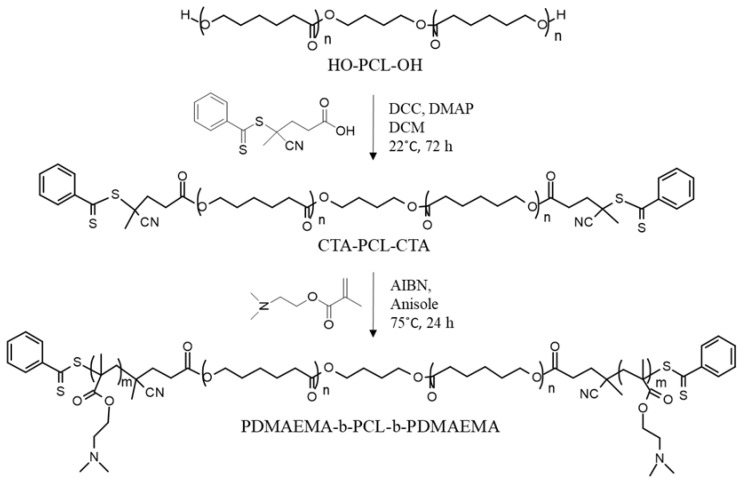
Synthesis of poly(2-(dimethylamino)ethyl methacrylate)-b-poly(ε-caprolactone)-b-poly(2-(dimethylamino)ethyl methacrylate) triblock copolymer via RAFT polymerization using a PCL-based bifunctional macro-CTA (CTA-PCL-CTA).

The PDMAEMA-b-PCL-b-PDMAEMA triblock copolymer was synthesized by reversible addition–fragmentation chain transfer (RAFT) polymerization. As shown in Figure 1, the synthesis procedure involves two stages. Firstly, PCL-diol was converted into a bifunctional poly(ε-caprolactone)-based macro chain transfer agent (CTA-PCL-CTA) via Steglich esterification of the terminal hydroxyl groups of PCL with carboxylic group from 4-Cyano-4-(phenylcarbonothioylthio)pentanoic acid in the presence of N,N’-dicyclohexylcarbodiimide (DCC) as coupling agent and 4-dimethylaminopyridine (DMAP) as a catalyst, in dry dichloromethane for 72 h. The prepared macro-RAFT agent was isolated and purified by precipitation in cold methanol. Next, DMAEMA was polymerized from CTA-PCL-CTA using AIBN as an initiator in anisole at 80 °C for 24 h, via the RAFT mechanism. After that, the polymerization was stopped, and the obtained copolymer was purified.

**Figure 2:**
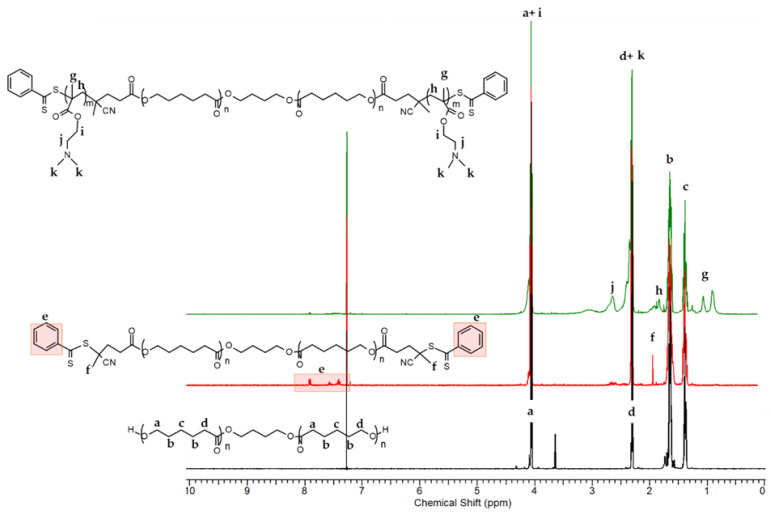
^1^H NMR spectra of PCL-diol, PCL-based macro-CTA (red) and PDMAEMA-b-PCL-b-PDMAEMA triblock copolymer (green) in CDCl3.

The PDMAEMA-b-PCL-b-PDMAEMA triblock copolymer was further characterized by ^1^H-NMR spectroscopy to confirm the chemical structures. The characteristic peaks are shown in Figure 2.

The main signals of the methylene protons from the PCL backbone at δ = 4.06–4.0 ppm (a), δ = 2.31 ppm (d), δ = 1.65 ppm (b), and δ = 1.39–1.35 ppm (c) are visible on the spectrum. Furthermore, the appearance of new signals (f) at δ = 1.85 ppm for the methyl groups of cyanopentanoic acid fragment and (e) at δ = 7.9–7.3 ppm from the phenyl protons from aromatics end groups indicates the success of the esterification reaction. Esterification degree of PCL-diol was calculated from the peak integral ratio of the methylene protons of PCL chain (-CH2, δ = 4.06 ppm, a) and aromatic protons (=CH-, δ = 7.3–7.9 ppm, e) of the phenyl group from cyano-4-(phenylcarbonothioylthio)pentanoic acid. The ^1^H NMR analysis proved that hydroxyl groups of the PCL were successfully esterified with CTA. The RAFT polymerization of DMAEMA afforded new intensive peaks at δ = 4.06–4.0 ppm (i) corresponding to the methylene protons from DMAEMA, and at δ = 2.45–2.31 ppm (k) to the methyl protons, which are overlayed with the methylene protons from PCL. New, less intense signals from the methylene protons (-CH_2_, in the interval δ = 2.53 ppm (j)) and methyl protons (-CH_3_, in the interval δ = 1.0–0.64 ppm, (g)) of the PDMAEMA segments were observed as well. The results revealed that the polymerization of DMAEMA proceeded with a conversion of approximately 75%. The calculated average degree of polymerization (DP) and molar mass of PDMAEMA-b-PCL-b-PDMAEMA triblock copolymer were 18 (2 × 9) and Mn = 6850 g/mol, respectively. The number-average molar masses and dispersity indices of the precursors, the macro-RAFT agent and the triblock copolymer, as calculated from the NMR and GPC analyses, are listed in Table 1.

### 3.2. Preparation and Characterization of PDMAEMA_9_-b-PCL_35_-b-PDMAEMA_9_ Micelles

**Figure 3:**
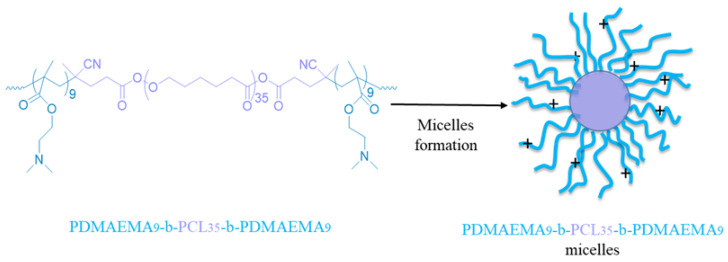
Schematic representation of the formation of cationic copolymer micellar nanocarriers via self-assembly of PDMAEMA_9_-b-PCL_35_-b-PDMAEMA_9_ triblock copolymer in water.

The cationic core–shell micellar nanocarriers were obtain by the self-assembly of the synthesized PDMAEMA_9_-b-PCL_35_-b-PDMAEMA_9_ triblock copolymer in aqueous media (Figure 3). The copolymer was dissolved in THF and then added dropwise to deionized water. Next, the organic solvents were evaporated to afford a stable colloidal solution (1 gL^−1^). The biodegradable and biocompatible PCL blocks of the copolymer formed the hydrophobic micelle core, whereas the hydrophilic PDMAEMA chains formed the cationic shell of the micelles.

**Figure 4:**
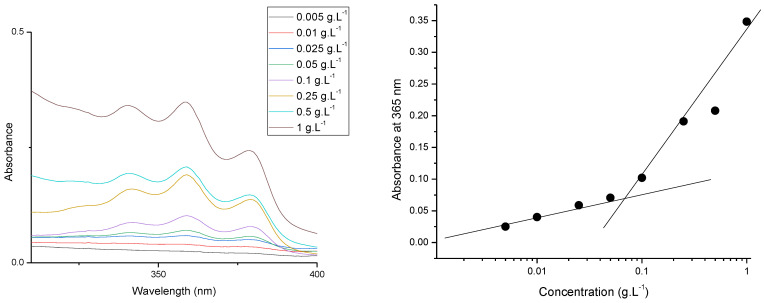
Determination of the critical micellar concentration of PDMAEMA–PCL–PDMAEMA triblock copolymer by the DPH method.

Firstly, the CMC of the synthesized amphiphilic block copolymer was determined spectrophotometrically by the DPH solubilizing method. The CMC of PDMAEMA_9_-b-PCL_35_-b-PDMAEMA_9_ triblock copolymer was 0.066 gL^−1^, as shown in Figure 4. Below the CMC, the micelles dissociate; the DPH spectrum, with a maximum at 356 nm, cannot be registered. Above the CMC, the formed micelles dissolved the hydrophobic dye and the intensity of characteristic DPH spectrum notably increased [7].

**Figure 5:**
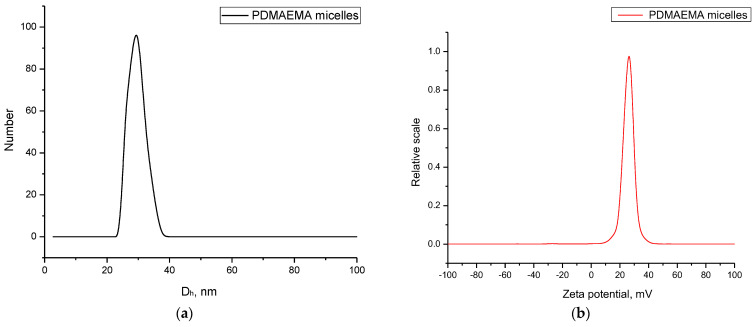
Hydrodynamic diameter (**a**) and zeta potential (**b**) of PDMAEMA_9_-b-PCL_35_-b-PDMAEMA_9_ micelles in water.

Next, the physicochemical characteristics of the obtained PDMAEMA micelles were investigated. The prepared carriers have monomodal particle size distribution with a hydrodynamic diameter (Dh) of approximately 31 nm (Figure 5a). The ELS measurement revealed a positive ζ-potential of 29 mV (Figure 5b). After dilution (from 1 gL^−1^ to 0.5 gL^−1^), the DLS and ELS measurements indicated that the micelles maintain an unchanged size and positive ζ-potential.

### 3.3. Preparation and Characterization of Complexes of PDMAEMA_9_-b-PCL_35_-b-PDMAEMA_9_ Micelles and SER

**Figure 6:**
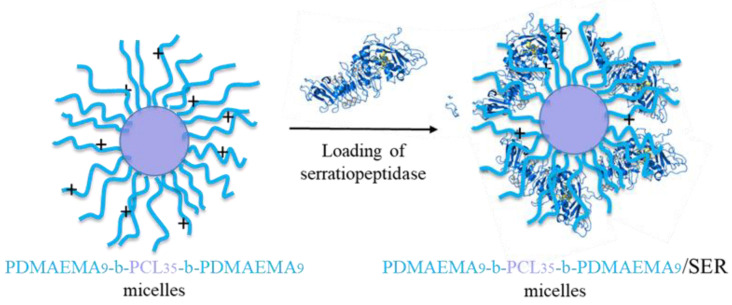
Schematic illustration of the complex formed by electrostatic interaction between serratiopeptidase molecules and PDMAEMA micellar shell.

Serratiopeptidase was embedded into the shell of polymeric micelles via reversibly physical absorption, involving H-bonding and electrostatic interactions between the positively charged PDMAEMA chains and the negatively charged enzyme molecules (Figure 6) [50]. Immobilization of enzymes by forming polyelectrolyte complexes is a widely used simple strategy based on the interactions between oppositely charged polyions [51].

**Figure 7:**
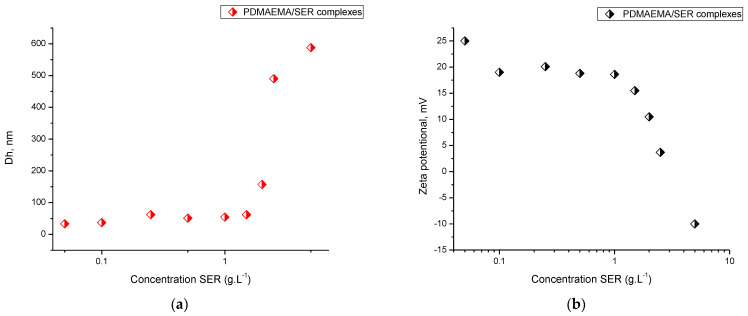
Hydrodynamic diameter (**a**) and zeta potential (**b**) of the PDMAEMA/SER complexes as a function of SER concentration.

As mentioned above, the PDMAEMA micelles have positive zeta potential of 29 mV, while SER has negative zeta potential of −22 mV, which implies successful formation of polyelectrolyte complexes. Nine series of complexes were prepared by adding different amounts of SER to the preformed micellar dispersion. The concentration of the micelles was kept constant (0.5 gL^−1^) for all investigated samples, and the concentration of SER varied from 0.05 to 5 gL^−1^. Firstly, the complexation process was studied by DLS and ELS measurements (see Figure 7 and Supplementary Materials). At low concentrations of SER (from 0.05 to 1 gL^−1^), the hydrodynamic diameter and ζ-potential of the obtained complexes were identical with the non-loaded PDMAEMA micelles (29 nm, 29 mV vs. 31 nm, 25 mV). A further increase in the enzyme concentration to 1.5 gL^−1^ led to a decrease in the ζ-potential to 15 mV, while the D_h_ of the particles increased to 60 nm. An additional increase in SER to 2.5 gL^−1^ resulted in a significant increase in particle size and a decrease in the ζ-potential to 3 mV. At the highest concentration of SER (5 gL^−1^), some agglomerates were observed. However, from the ELS measurement, we did not observe a second peak corresponding to the free enzyme in the solution. Most likely, at this concentration, SER crosslinks the PDMAEMA micelles through intra-micellar electrostatic interactions.

**Figure 8:**
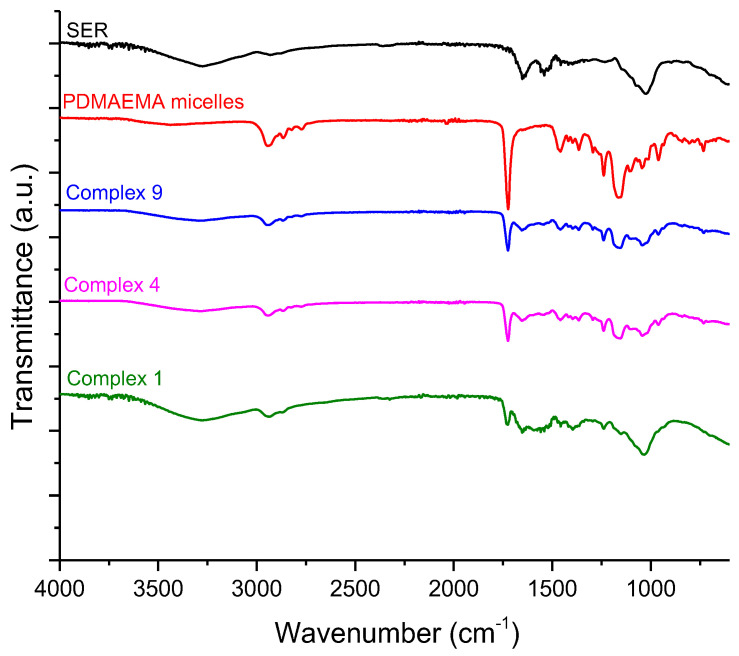
FTIR spectra of pure SER (black line), blank micelles (red line) and PDMAEMA/SER complexes at various concentrations of SER (0.05 gL^−1^—blue line; 1.5 gL^−1^—pink line; 5 gL^−1^—green line).

The incorporation of SER into polymeric micelles was confirmed by FTIR analysis. The FTIR spectrum of the freeze-dried samples of PDMAEMA micelles, SER, and PDMAEMA/SER complexes (1:0.1—complex 9; 1:3—complex 4; 1:10—complex 1) are shown in Figure 8. The well-defined and sharp peak at 1723 cm^−1^ is attributed to the stretching vibrations of C=O groups of esters from PCL and PDMAEMA. The main characteristic peaks of the PDMAEMA micelles were observed at approximately 2940 cm^−1^ (C–H bond), 2860 cm^−1^, and 2770 cm^−1^ (also the C–H bond, but for the N(CH_3_)_2_ group from PDMAEMA); the stretching vibrations of C–O–C at 1238 and a band from the stretching vibrations of the C–N and C–O groups had a maximum at 1162 cm^−1^.

The spectrum of serratiopeptidase showed characteristic absorption bands at 3500–3000 cm^−1^ (O–H and N–H bonds), and at 2920 cm^−1^, corresponding to the vibrations of the C–H bond of the alkyl chains. The strong bands at approximately 1652 cm^−1^ and 1540 cm^−1^ belong to the amide linkages of the enzyme (C=O bending vibration of amide I and N–H and stretching of amide II, respectively). After complexation, the main peaks of PDMAEMA micelles and serratiopeptidase were observed on the FTIR spectrum. The intensity of the peaks at 2860 cm^−1^ and 2770 cm^−1^, related to the vibration of C–H bonds existing in –N(CH_3_)_2_ groups of PDMAEMA chains, decreased with the increase in SER concentration. The intensity of C=O stretching vibrations was considerably suppressed with the increase in enzyme into the complexes as well. Comparing the FTIR spectra of PDMAEMA/SER complexes with that of the pure enzyme, no significant changes in the characteristic bands of the protein were observed, which indicates that SER involved in the complex has not undergone changes in its secondary structure. A decrease in the intensity at 1723 cm^−1^ and 1540 cm^−1^ could be due to hydrogen bonds formed by C=O group of PDMAEMA and the N–H group of SER [52,53].

The immobilization of enzymes in cationic micelles might induce changes in enzyme activity [20]. In our case, the proteolytic activity of SER was determined in the concentration interval 0.05 g·L^−1^ and 0.1 g·L^−1^, before and after loading into the polymeric micelles. The assay is based on proteolytic hydrolysis of casein at pH 7.0 and 37 °C, for 30 min, followed by a determination of the solubilized casein (tyrosine) by spectrophotometry. The results (Table 2) revealed that SER, loaded into polymeric micelles, maintained its activity. We should mention that, at a higher SER concentration, the absorbance of tyrosine becomes very high (cannot be recorded), which is a limit of the method.

### 3.4. In Vitro Release Study

**Figure 9:**
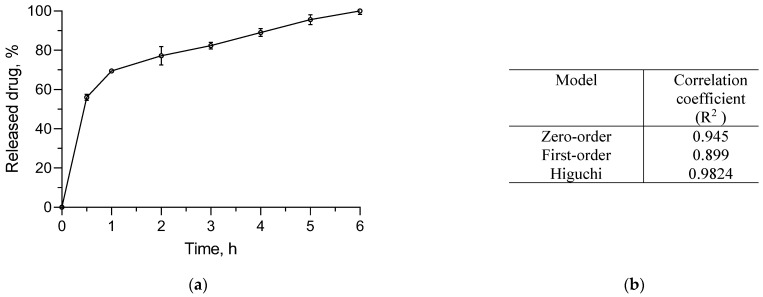
In vitro release of serratiopeptidase (1 mg/mL) from the complex in phosphate buffer (pH of 7.4) (**a**) and kinetic analysis of the data (**b**).

The in vitro release test was performed in a buffer with a pH of 7.4. This pH value was selected with the consideration that the pH value of damaged skin is higher than 5 [54,55]. The results showed a strongly expressed burst effect in the first 30 min: almost 60% of the enzyme was released (Figure 9). The probable explanation for this initial burst release could be the high aqueous solubility of serratiopeptidase and its location in the shell of the micelles. After the initial release, the complete release of the enzyme occurred in a sustained manner for 6 h. The fitting of the release data after the initial burst suggested that the process was controlled by diffusion. Thus, the sustained release could be considered an appropriate achievement for more effective wound healing. Moreover, the sustained release of the enzyme would improve microcirculation, which is known to enable the wound-healing process [56].

### 3.5. Cytotoxicity and Wound Healing

**Figure 10:**
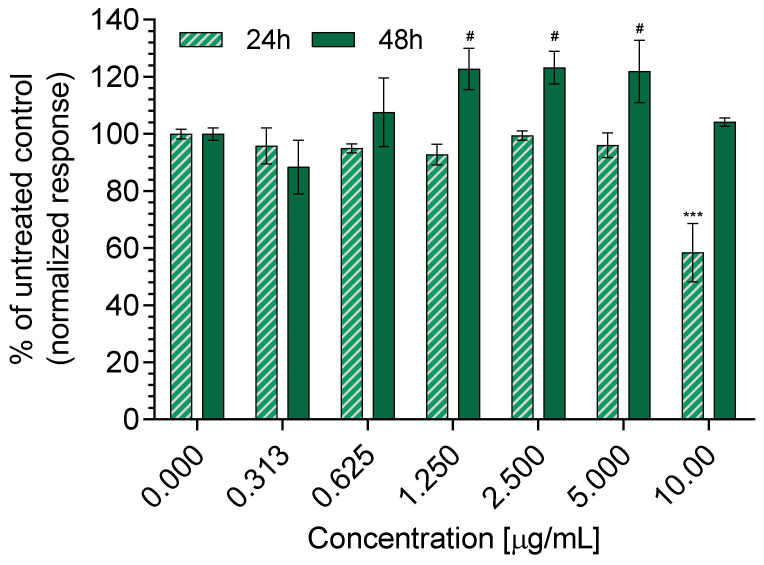
In vitro cytotoxicity assay of serratiopeptidase complexes on HaCaT cells for 24 and 48 h; *** *p* < 0.001 vs. control group at 24 h, # *p* < 0.05 vs. control group at 48 h.

To evaluate the cytotoxic potential of the serratiopeptidase complexes, we conducted MTT tests on the healthy keratinocytes of HaCaT after 24 and 48 h of treatment with the complexes in the 0.313–10 µg/mL concentration range (Figure 10). The results showed that the enzyme reduces the viability of the cells at 10 µg/mL when applied for 24 h. All other concentrations did not show cytotoxic effects. For 48 h of treatment, no toxic effect was measured. This can be explained with the ability of cells to adapt to toxicity stress, for example, via mitochondrial biogenesis [57]. More importantly, for 48 h of treatment, a significant increase in the cell viability was observed at 1.25, 2.5, and 5 µg/mL concentrations. These results revealed the ability of the systems to enhance the proliferation of the keratinocytes, which is vastly beneficial for wound healing. Keratinocytes are known to stimulate the production of growth factors from fibroblasts, inducing exogenous pathogens destruction and immune cell activation [15,58,59]. Therefore, we chose these three concentrations for the next experiments.

**Figure 11:**
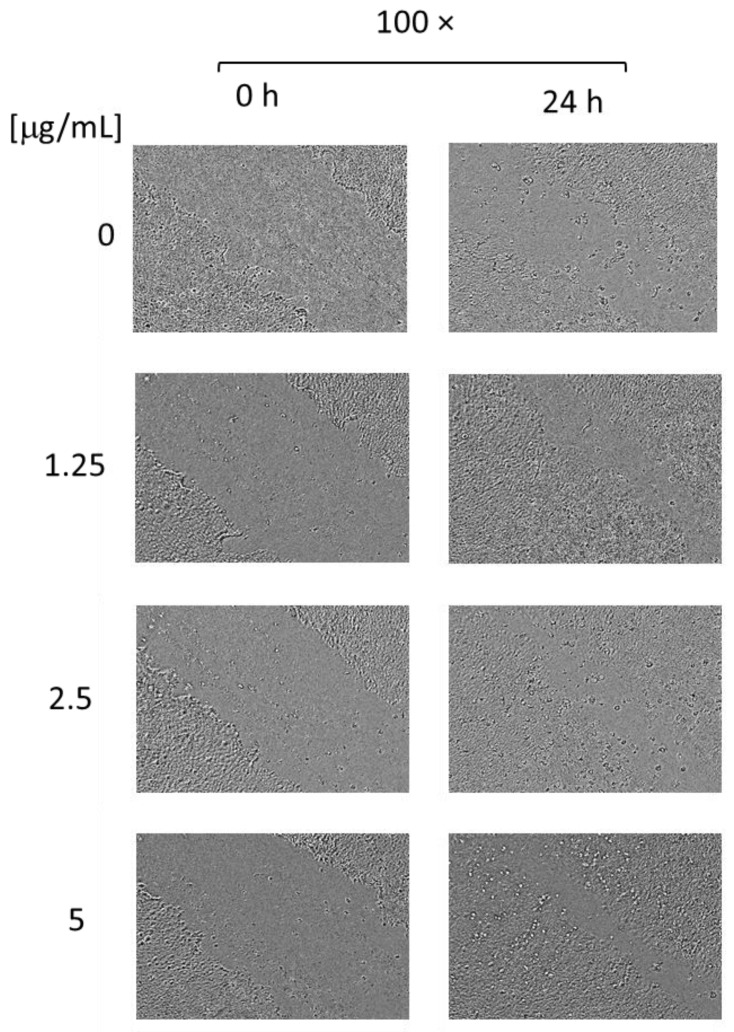
Digital images made 24 h after the scratch of non-treated (0 µg/mL) HaCaT cells and HaCaT cells treated with the complexes (1.25, 2.5, and 5 µg/mL).

**Figure 12:**
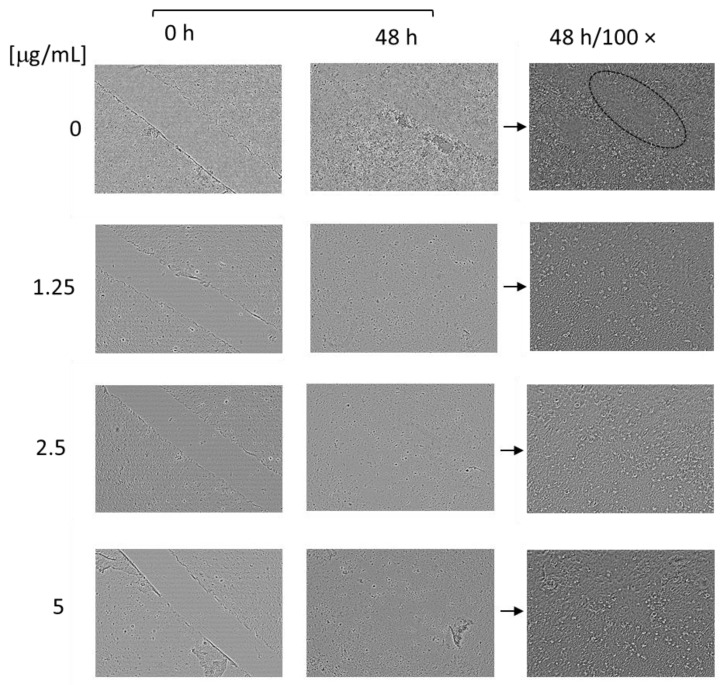
Digital images made 48 h after the scratch of non-treated (0 µg/mL) HaCaT cells and HaCaT cells treated with the complexes (1.25, 2.5, and 5 µg/mL).

The proteolytic enzyme possesses the ability to degrade dead tissue, proteins, and abnormal exudates and to enhance the blood and lymph absorption of fragmented products, leading to a potential wound-healing effect. Moreover, the enzyme has anti-inflammatory, antibacterial, anti-oedemic, and analgesic effects [12,31,60,61,62]. Therefore, aiming to confirm the potential of the complexes to enhance migration and proliferation in vitro, we conducted scratch assays on two cell lines: human keratinocytes HaCaT and human gingival fibroblasts HGF. The results from the test on HaCaT cells revealed that the nanosized systems have a positive effect on the closing rate at all three concentrations (Figure 11 and Figure 12). After 24 h of treatment, the closure rate for the control cells was 33.45%; for the cells treated with the complexes at concentrations of 1.25, 2.5, or 5 µg/mL, the closure rates were 68.62%, 69.41%, and 73.4%, respectively. Moreover, after 48 h of treatment, the scratch area was completely closed in all of the treated groups (Figure 12). However, in the control group, which was not treated with the complexes, there was still a gap uncovered with cells (Figure 12, the area enclosed by an ellipse). Therefore, the SER complexes significantly enhanced the migration and proliferation of the keratinocytes, especially at the highest concentration.

**Figure 13:**
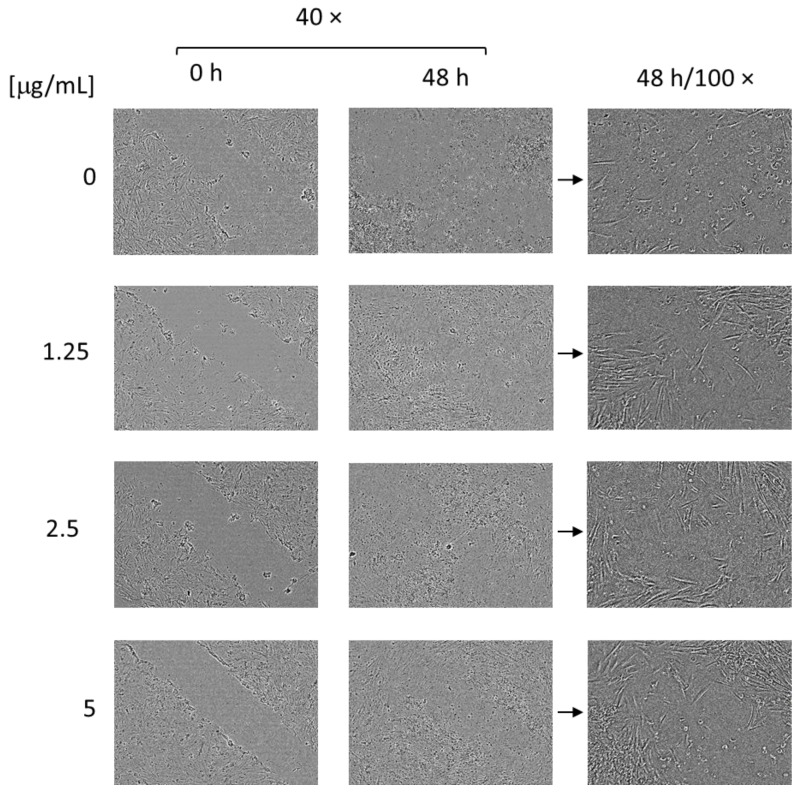
Digital images made 48 h after the scratch of non-treated (0 µg/mL) HGF cells and HGF cells treated with the complexes (1.25, 2.5, and 5 µg/mL).

The results from the assay conducted on the gingival fibroblasts, HGF, revealed that the complexes had a concentration-dependent enhancing effect on proliferation and migration (Figure 13). In particular, after 48 h of treatment, the closure rate for non-treated cells was 12.77%; for the cells treated with the complexes at concentrations of 1.25, 2.5, or 5 µg/mL, the closure rates were 18.84%, 50.82%, and 71.41%, respectively. These data confirm the significant potential of the enzyme to improve the migration and proliferation of fibroblast cells, too. It is important to note that fibroblasts are a key factor for the contraction of wounds; they participate in the degradation of fibrin clots and in the creation of collagen structures and new extra cellular matrices [63,64].

## 4. Conclusions

Cationic block copolymer micelles based on a well-defined amphiphilic PDMAEMA_9_-b-PCL_35_-b-PDMAEMA_9_ triblock copolymer were developed as carriers of the proteolytic enzyme serratiopeptidase. SER-loaded nanosized particles were successfully obtained by electrostatic interaction between the positively charged PDMAEMA micellar shell and negatively charged SER. The incorporation of SER into the polymeric micelles was confirmed by FTIR and ELS analysis. At low SER concentrations (from 0.05 to 1 gL^−1^), the hydrodynamic diameter and ζ-potential of the obtained complexes were similar to the blank micelles; meanwhile, at high enzyme concentrations (≥1.5 gL^−1^), some larger particles and precipitations were detected. The resulting complexes did not affect the high proteolytic activity of the enzyme and exhibited a concentration-dependent enhancing effect on the proliferation and migration of human keratinocyte and gingival fibroblast cells. These findings give us a reason to consider the newly developed complexes as promising candidates for applications in wound healing.

## Figures and Tables

**Table 1:** Composition and molecular characteristics of: HO-PCL-OH precursor, macro-CTA, and PDMAEMA-b-PCL-b-PDMAEMA triblock copolymer.

Sample Code	M_n_^NMR^ g mol^−1^	M_n_^GPC^ g mol^−1^	M_w_/M_n_
HO-PCL_35_-OH	4000	6290	1.54
CTA-PCL_35_-CTA	4550	8402	1.56
PDMAEMA_9_-b-PCL_35_-b-PDMAEMA_9_	6820	15000	1.32

**Table 2:** Proteolytic activity of SER before and after complexation with the cationic micelles, at two concentrations.

SER g/L	Activity of Free SER U/mL	Activity of PDMAEMA/SER Complex U/mL
0.05	1.31	1.46
0.1	2.15	1.98

## Data Availability

The data presented in this study are openly available in the article.

## Supplementary Materials

The following supporting information can be downloaded at: https://www.mdpi.com/article/10.3390/pharmaceutics16080988/s1, Figure S1: Size distribution plots of complexes of PDMAEMA micelles with serratiopeptidase obtained at different mass ratio polymer/enzyme (from 1:0.1 to 1:10): (a) complex 9 (0.05 gL^−1^ SER) (b) complex 8 (0.1 gL^−1^ SER); (c) complex 7 (0.25 gL^−1^ SER); (d) complex 6 (0.5 gL^−1^ SER); (e) complex 5 (1 gL^−1^ SER); (f) complex 4 (1.5 gL^−1^ SER); (g) complex 3 (2 gL^−1^ SER); (h) complex 2 (2.5 gL^−1^ SER); (i) complex 1 (5 gL^−1^ SER); Figure S2: Zeta potential of: (a) serratiopeptidase and complexes of PDMAEMA micelles with serratiopeptidase obtained at different mass ratio polymer/enzyme (from 1:0.1 to 1:10): (b) complex 9 (0.05 gL^−1^ SER) (c) complex 8 (0.1 gL^−1^ SER); (d) complex 7 (0.25 gL^−1^ SER); (e) complex 6 (0.5 gL^−1^ SER); (f) complex 5 (1 gL^−1^ SER); (g) complex 4 (1.5 gL^−1^ SER); (h) complex 3 (2 gL^−1^ SER); (i) complex 2 (2.5 gL^−1^ SER); (j) complex 1 (5 gL^−1^ SER).

## References

[1] Choi Y.H., Han H.K. (2018). Nanomedicines: Current status and future perspectives in aspect of drug delivery and pharmacokinetics. J. Pharm. Investig..

[2] De R., Mahata M.K., Kim K.-T. (2022). Structure-Based Varieties of Polymeric Nanocarriers and Influences of Their Physicochemical Properties on Drug Delivery Profiles. Adv. Sci..

[3] Halwani A.A. (2022). Development of Pharmaceutical Nanomedicines: From the Bench to the Market. Pharmaceutics.

[4] Avramović N., Mandić B., Savić-Radojević A., Simić T. (2020). Polymeric Nanocarriers of Drug Delivery Systems in Cancer Therapy. Pharmaceutics.

[5] Vardaxi A., Kafetzi M., Pispas S. (2022). Polymeric Nanostructures Containing Proteins and Peptides for Pharmaceutical Applications. Polymers.

[6] Borandeh S., van Bochove B., Teotia A., Seppälä J. (2021). Polymeric drug delivery systems by additive manufacturing. Adv. Drug Deliv. Rev..

[7] Ghezzi M., Pescina S., Padula C., Santi P., Del Favero E., Cantù L., Nicoli S. (2021). Polymeric micelles in drug delivery: An insight of the techniques for their characterization and assessment in biorelevant conditions. J. Control. Release.

[8] Marras A.E., Ting J.M., Stevens K.C., Tirrell M.V. (2021). Advances in the Structural Design of Polyelectrolyte Complex Micelles. J. Phys. Chem..

[9] Uchida S., Kataoka K. (2019). Design concepts of polyplex micelles for in vivo therapeutic delivery of plasmid DNA and messenger RNA. J. Biomed. Mater. Res..

[10] de la Fuente M., Lombardero L., Gómez-González A., Solari C., Angulo-Barturen I., Acera A., Vecino E., Astigarraga E., Barreda-Gómez G. (2021). Enzyme Therapy: Current Challenges and Future Perspectives. Int. J. Mol. Sci..

[11] Vellard M. (2003). The enzyme as drug: Application of enzymes as pharmaceuticals. Curr. Opin. Biotechnol..

[12] Tiwari M. (2017). The role of serratiopeptidase in the resolution of inflammation. Asian J. Pharm. Sci..

[13] Verma M.K., Pulicherla K.K. (2016). Enzyme promiscuity in Earthworm serine protease-Substrate versatility and therapeutic potential. Amino Acids.

[14] Li Y., Cirino P.C. (2014). Recent advances in engineering proteins for biocatalysis. Biotechnol. Bioeng..

[15] Jadhav S.B., Shah N., Rathi A., Rathi V., Rathi A. (2020). Serratiopeptidase: Insights into the therapeutic applications. Biotechnol. Rep..

[16] Patel V.R., Agarwal Y.K. (2011). Nanosuspension: An approach to enhance solubility of drug. J. Adv. Pharm. Technol. Res..

[17] Mohamad N.R., Marzuki N.H., Buang N.A., Huyop F., Wahab R.A. (2015). An overview of technologies for immobilization of enzymes and surface analysis techniques for immobilized enzymes. Biotechnol. Biotechnol. Equip..

[18] Brena B., González-Pombo P., Batista-Viera F. (2013). Immobilization of enzymes: A literature survey. Methods Mol. Biol..

[19] Maghraby Y., El-Shabasy R., Ibrahim A., Azzazy H. (2023). Enzyme Immobilization Technologies and Industrial Applications. ACS Omega.

[20] Muronetz V.I., Pozdyshev D.V., Semenyuk P.I. (2022). Polyelectrolytes for Enzyme Immobilization and the Regulation of Their Properties. Polymers.

[21] Dong Z., Wei H., Mao J., Wang D., Yang M., Bo S., Ji X. (2012). Synthesis and responsive behavior of poly(N,N-dimethylaminoethyl methacrylate) brushes grafted on silica nanoparticles and their quaternized derivatives. Polymer.

[22] Schepelina O., Zharov I. (2008). Poly(2-(dimethylamino)ethyl methacrylate)-modified nanoporous Colloidal films with pH and ion response. Langmuir.

[23] Stawski D. (2023). Poly(N,N-dimethylaminoethyl methacrylate) as a bioactive polyelectrolyte-production and properties. R. Soc. Open Sci..

[24] Lei H., Wang M., Tang Z., Luan Y., Liu W., Song B., Chen H. (2013). Control of Lysozyme Adsorption by pH on Surfaces Modified with Polyampholyte Brushes. Langmuir.

[25] Ganguli S., Yoshimoto K., Tomita S., Sakuma H., Matsuoka T., Shiraki K., Nagasaki Y. (2009). Regulation of lysozyme activity based on thermotolerant protein/smart polymer complex formation. J. Am. Chem. Soc..

[26] Haladjova E., Kyulavska M., Doumanov J., Topouzova-Hristova T., Petrov P. (2017). Polymeric vehicles for transport and delivery of DNA via cationic micelle template method. Colloid. Polym. Sci..

[27] Kamenova K., Haladjova E., Grancharov G., Kyulavska M., Tzankova V., Aluani D., Yoncheva K., Pispas S., Petrov P. (2018). Co-assembly of block copolymers as a tool for developing novel micellar carriers of insulin for controlled drug delivery. Eur. Polym. J..

[28] Sentoukas T., Pispas S. (2020). Poly(2-(dimethylamino)ethyl methacrylate)-b-poly (hydroxypropyl methacrylate) copolymers/bovine serum albumin complexes in aqueous solutions. J. Polym. Sci..

[29] Sentoukas T., Pispas S. (2020). Poly(2-[dimethylamino]ethyl methacrylate)-b-poly (hydroxypropyl methacrylate)/DNA polyplexes in aqueous solutions. J. Polym. Sci..

[30] Gupte V., Luthra U. (2017). Analytical techniques for serratiopeptidase: A review. J. Pharm. Anal..

[31] Nair S.R. (2022). Serratiopeptidase: An integrated View of Multifaceted Therapeutic Enzyme. Biomolecules.

[32] Maeda H., Morihara K. (1995). Serralysin and related bacterial proteinases. Methods Enzymol..

[33] Bhagat S., Agarwal M., Roy V. (2013). Serratiopeptidase: A systematic review of the existing evidence. Int. J. Surg..

[34] Mali N., Wavikar P., Vavia P. (2015). Serratiopeptidase loaded chitosan nanoparticles by polyelectrolyte complexation: In vitro and in vivo evaluation. AAPS PharmSciTech..

[35] Maejima K., Miyata K., Tomoda K. (1983). A Manganese Superoxide Dismutase from *Serratia marcescens*. Agric. Biol. Chem..

[36] Santhosh K. (2018). The emerging role of serratiopeptidase in oral surgery: Literature update. Asian J. Clin. Pharm. Res..

[37] Jadav S.P., Patel N.H., Shah T.G., Gajera M.V., Trivedi H.R., Shah B.K. (2010). Comparison of anti-inflammatory activity of serratiopeptidase and diclofenac in albino rats. J. Pharmacol. Pharmacother..

[38] Sharm C., Jha N.K., Meeran M.F.N., Patil C.R., Goyal S.N., Ojha S. (2021). Serratiopeptidase, a Serine Protease Anti-Inflammatory, Fibrinolytic, and Mucolytic Drug, Can Be a Useful Adjuvant for Management in COVID-19. Front. Pharmacol..

[39] Kumar S., Jana A.K., Dhamija I., Singla Y., Maiti M. (2013). Preparation, characterization and targeted delivery of serratiopeptidase immobilized on amino-functionalized magnetic nanoparticles. Eur. J. Pharm. Biopharm..

[40] Kaur H., Singh A. (2015). Design, development and characterization of serratiopeptidase loaded albumin nanoparticles. J. App. Pharm. Sci..

[41] Singh D., Singh M.R. (2012). Development of antibiotic and debriding enzyme-loaded PLGA microspheres entrapped in PVA-gelatin hydrogel for complete wound management. Artif. Cell. Blood Sub. Biotechnol..

[42] Hire N.N., Deore A.B., Derle D.V., Nathe R. (2014). Formulation and evaluation of serratiopeptidase microspheres using eudragit rs100 polymer. World J. Pharm..

[43] Sandhya K.V., Devi G.S., Mathew S.T. (2008). Liposomal formulations of serratiopeptidase: In vitro studies using PAMPA and Caco-2 models. Mol. Pharm..

[44] Cupp-Enyard C. (2008). Sigma’s Non-specific Protease Activity Assay—Casein as a Substrate. J. Vis. Exp..

[45] (2009). In Vitro Cytotoxicity of Medical Devices.

[46] Mosmann T. (1983). Rapid Colorimetric Assay for Cellular Growth and Survival: Application to Proliferation and Cytotoxicity Assays. J. Immunol. Methods.

[47] Sung T.-J., Wang Y.-Y., Liu K.-L., Chou C.-H., Lai P.-S., Hsieh C.-W. (2020). Pholiota Nameko Polysaccharides Promotes Cell Proliferation and Migration and Reduces ROS Content in H_2_O_2_-Induced L929 Cells. Antioxidants.

[48] Walter M.N.M., Wright K.T., Fuller H.R., MacNeil S., Johnson W.E.B. (2010). Mesenchymal Stem Cell-Conditioned Medium Accelerates Skin Wound Healing: An in Vitro Study of Fibroblast and Keratinocyte Scratch Assays. Exp. Cell Res..

[49] Collins T.J. (2007). ImageJ for Microscopy. BioTechniques.

[50] Petrila L.-M., Grădinaru V.R., Bucatariu F., Mihai M. (2022). Polymer/Enzyme Composite Materials—Versatile Catalysts with Multiple Applications. Chemistry.

[51] Lankalapalli S., Kolapalli V.R. (2009). Polyelectrolyte Complexes: A Review of their Applicability in Drug Delivery Technology. Indian. J. Pharm. Sci..

[52] Blažic R., Kučić Grgić D., Kraljić Roković M., Vidović E. (2022). Cellulose-g-poly(2-(dimethylamino)ethylmethacrylate) Hydrogels: Synthesis, Characterization, Antibacterial Testing and Polymer Electrolyte Application. Gels.

[53] Fotaki D., Karayianni M., Skandalis A., Haladjova E., Forys A., Trzebicka B., Rangelov S., Pispas S. (2024). Complexation of poly(methacrylic acid) star polyelectrolytes with lysozyme. Eur. Polym. J..

[54] Segger D., Aßmus U., Brock M., Erasmy J., Finkel P., Fitzner A., Heuss H., Kortemeier U., Munke S., Rheinländer T. (2008). Multicenter Study on Measurement of the Natural PH of the Skin Surface. Int. J. Cosmet. Sci..

[55] Kuo S.-H., Shen C.-J., Shen C.-F., Cheng C.-M. (2020). Role of pH Value in Clinically Relevant Diagnosis. Diagnostics.

[56] Strodtbeck F. (2001). Physiology of wound healing. Newborn Infant. Nurs. Rev..

[57] Kwan P., Desmoulière A., Tredget E.E. (2018). 45—Molecular and Cellular Basis of Hypertrophic Scarring. Total Burn Care.

[58] Piipponen M., Li D., Landén N.X. (2020). The Immune Functions of Keratinocytes in Skin Wound Healing. Int. J. Mol. Sci..

[59] Werner S., Krieg T., Smola H. (2007). Keratinocyte–Fibroblast Interactions in Wound Healing. J. Investig. Dermatol..

[60] Kumar D., Verma D., Abbot V. (2023). A Review on Pharmaceutical, Pharmacological and Chemical Aspects of Serratiopeptidase as Anti-Inflammatory Agent. Mater Today Proc..

[61] Chandrasekaran S.D., Selvakumar J.N., Vaithilingam M. (2019). Serratiopeptidase: A Multifaceted Microbial Enzyme in Health Care. Biotechnology of Microorganisms.

[62] Rouhani M., Valizadeh V., Bakhshandeh H., Hosseinzadeh S.A., Molasalehi S., Atyabi S.M., Norouzian D. (2023). Improved Anti-Biofilm Activity and Long-Lasting Effects of Novel Serratiopeptidase Immobilized on Cellulose Nanofibers. Appl. Microbiol. Biotechnol..

[63] Bainbridge P. (2013). Wound Healing and the Role of Fibroblasts. J. Wound Care.

[64] desJardins-Park H.E., Foster D.S., Longaker M.T. (2018). Fibroblasts and Wound Healing: An Update. Regen. Med..

