# Supplementary material for: Nanosized Complexes of the Proteolytic Enzyme Serratiopeptidase with Cationic Block Copolymer Micelles Enhance the Proliferation and Migration of Human Cells
Source: Pharmaceutics. 2024 Jul 25;16(8):988. doi: 10.3390/pharmaceutics16080988 (PMC11358905; doi:10.3390/pharmaceutics16080988)
Supplement: Supplementary file 1 [file pharmaceutics-16-00988-s001.zip › pharmaceutics-3113682-supplementary.pdf]

pharmaceutics

Supplementary Materials:

Nanosized complexes of the proteolytic enzyme serratiopeptidase with cationic block copolymer micelles enhance the proliferation and migration of human cells

Katya Kamenova[1], Anna Prancheva[1], Lyubomira Radeva[2], Krassimira Yoncheva[2], Maya M. Zaharieva[3], Hristo M. Najdenski[3], Petar D. Petrov[1,*]

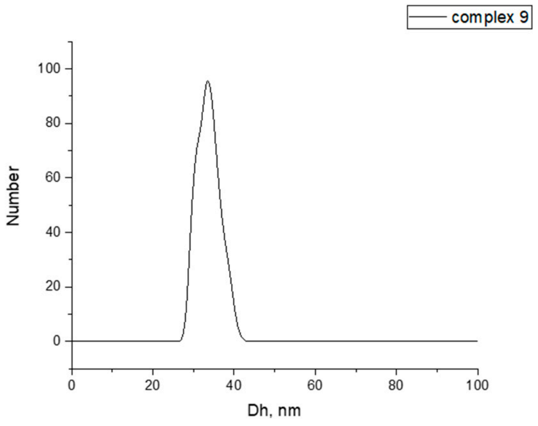

(a)

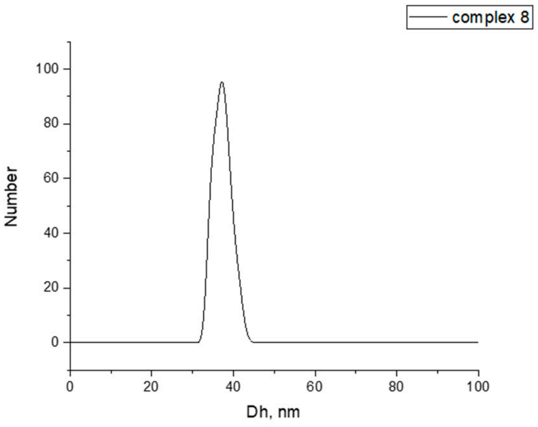

(b)

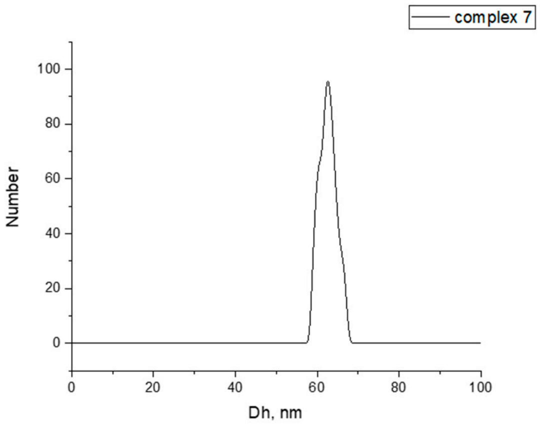

(c)

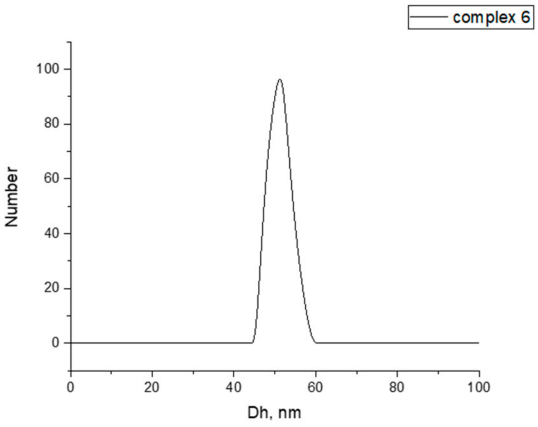

(d)

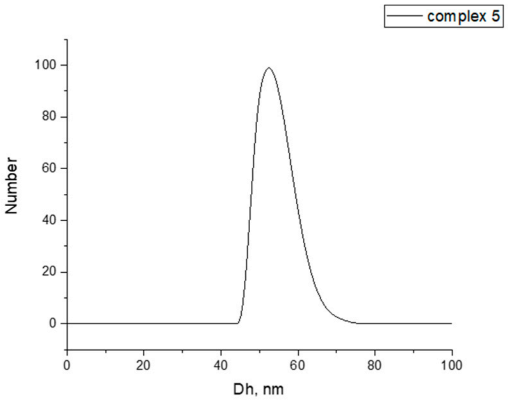

(e)

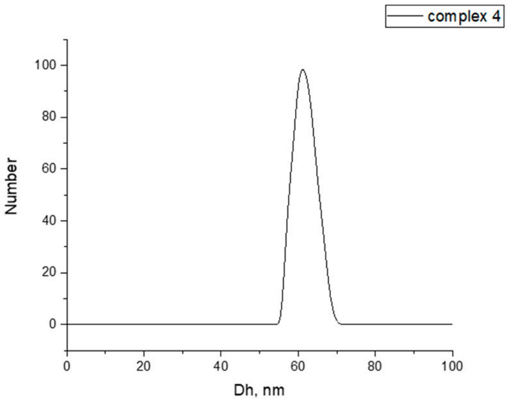

(f)

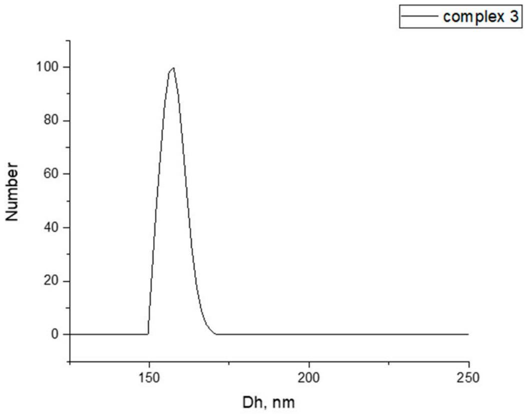

(g)

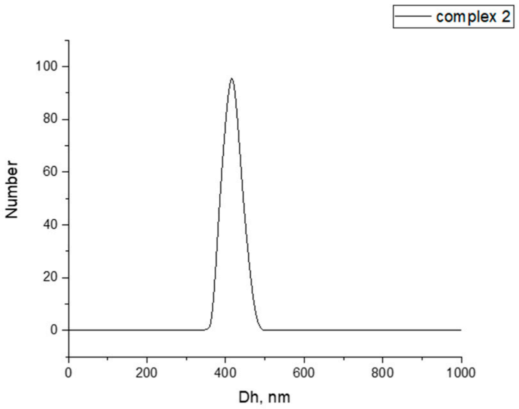

(h)

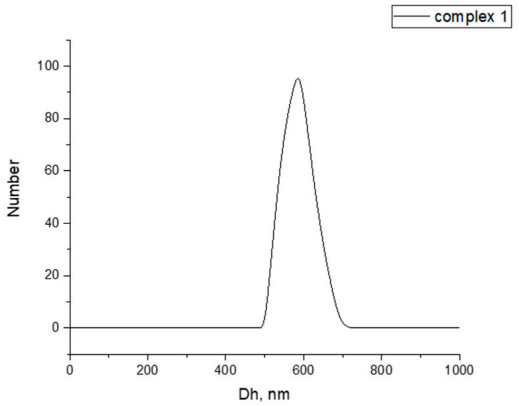

(i)

Figure S1. Size distribution plots of complexes of PDMAEMA micelles with serratiopeptidase obtained at different mass ratio polymer/enzyme (from 1:0.1 to 1:10): (**a**) complex 9 (0.05 gL^{-1} SER) (**b**) complex 8 (0.1 gL^{-1} SER); (**c**) complex 7 (0.25 gL^{-1} SER); (**d**) complex 6 (0.5 gL^{-1} SER); (**e**) complex 5 (1 gL^{-1} SER); (**f**) complex 4 (1.5 gL^{-1} SER); (**g**) complex 3 (2 gL^{-1} SER); (**h**) complex 2 (2.5 gL^{-1} SER); (**i**) complex 1 (5 gL^{-1} SER).

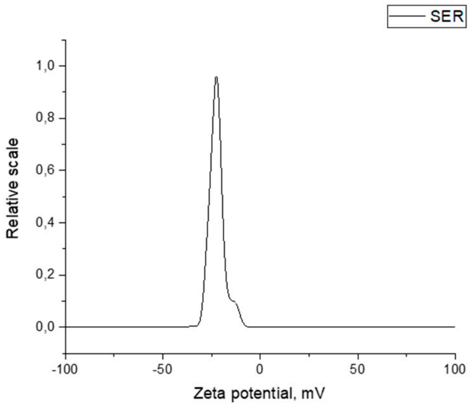
SER
Relative scale
1,0
0,8
0,6
0,4
0,2
0,0
-100
-50
0
50
100
Zeta potential, mV

(**a**)

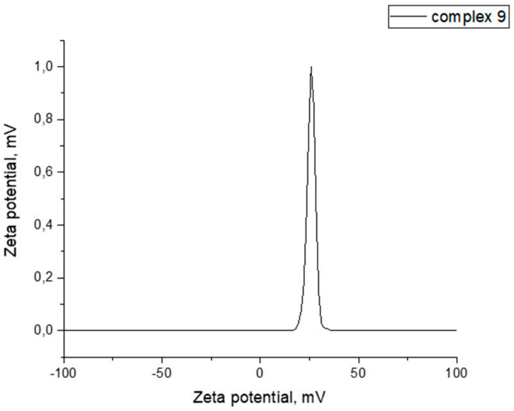
complex 9
Zeta potential, mV
1,0
0,8
0,6
0,4
0,2
0,0
-100
-50
0
50
100
Zeta potential, mV

(**b**)

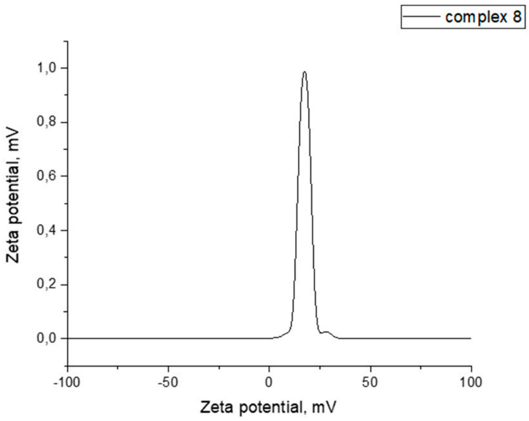
complex 8
Zeta potential, mV
1,0
0,8
0,6
0,4
0,2
0,0
-100
-50
0
50
100
Zeta potential, mV

(**c**)

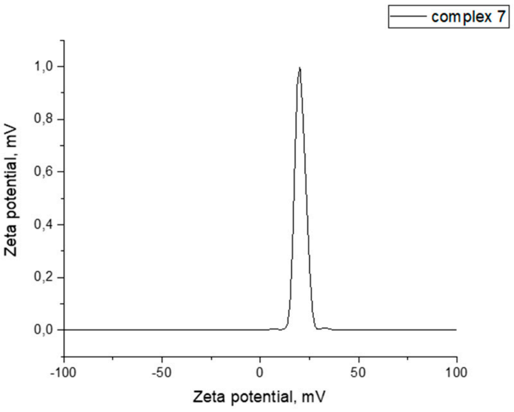
complex 7
Zeta potential, mV
1,0
0,8
0,6
0,4
0,2
0,0
-100
-50
0
50
100
Zeta potential, mV

(**d**)

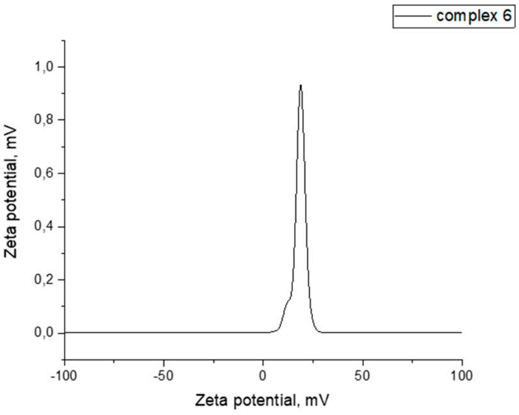
complex 6
Zeta potential, mV
1,0
0,8
0,6
0,4
0,2
0,0
-100
-50
0
50
100
Zeta potential, mV

(**e**)

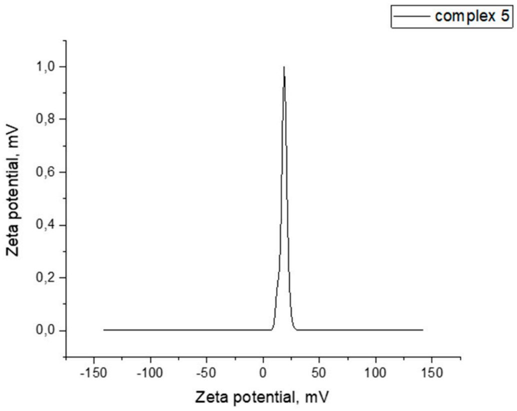
complex 5
Zeta potential, mV
1,0
0,8
0,6
0,4
0,2
0,0
-150
-100
-50
0
50
100
150
Zeta potential, mV

(**f**)

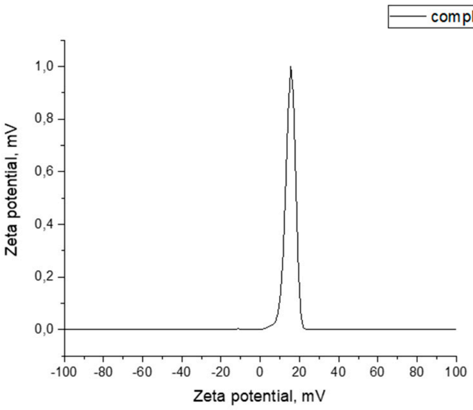

(g)

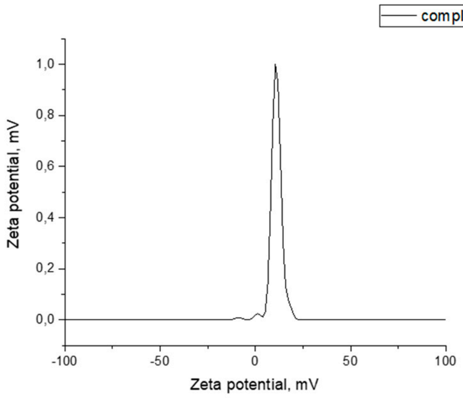

(h)

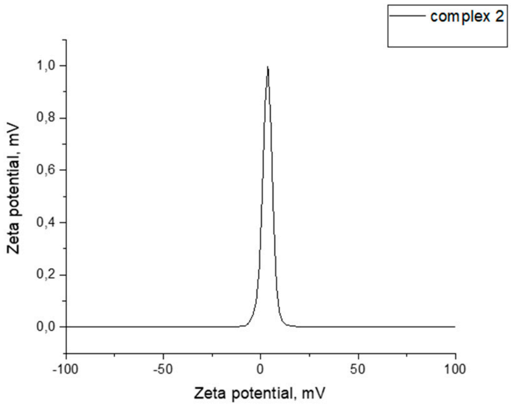

(i)

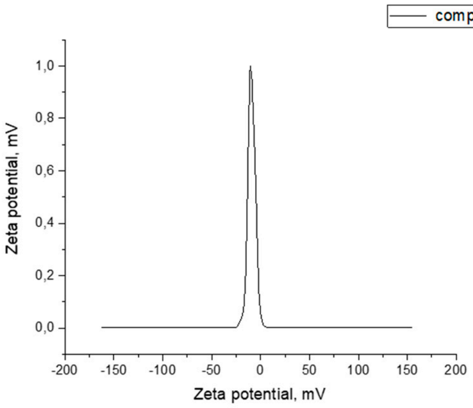

(j)

Figure S2. Zeta potential of: (**a**) serratiopeptidase and complexes of PDMAEMA micelles with serratiopeptidase obtained at different mass ratio polymer/enzyme (from 1:0.1 to 1:10): (**b**) complex 9 (0.05 gL^{-1} SER) (**c**) complex 8 (0.1 gL^{-1} SER); (**d**) complex 7 (0.25 gL^{-1} SER); (**e**) complex 6 (0.5 gL^{-1} SER); (**f**) complex 5 (1 gL^{-1} SER); (**g**) complex 4 (1.5 gL^{-1} SER); (**h**) complex 3 (2 gL^{-1} SER); (**i**) complex 2 (2.5 gL^{-1} SER); (**j**) complex 1 (5 gL^{-1} SER).
